# First phylogenetic analysis of Dryophthorinae (Coleoptera, Curculionidae) based on structural alignment of ribosomal DNA reveals Cenozoic diversification

**DOI:** 10.1002/ece3.7131

**Published:** 2021-02-09

**Authors:** Maria Lourdes Chamorro, Bruno A. S. de Medeiros, Brian D. Farrell

**Affiliations:** ^1^ Systematic Entomology Laboratory ARS, USDA, c/o National Museum of Natural History Washington DC USA; ^2^ Smithsonian Tropical Research Institute Panama City Panama; ^3^ Department of Organismic and Evolutionary Biology Harvard University Cambridge MA USA

**Keywords:** angiosperms, coevolution, fossilized birth‐death model, node dating, palm weevils, red palm weevil, structural alignment

## Abstract

Dryophthorinae is an economically important, ecologically distinct, and ubiquitous monophyletic group of pantropical weevils with more than 1,200 species in 153 genera. This study provides the first comprehensive phylogeny of the group with the aim to provide insights into the process and timing of diversification of phytophagous insects, inform classification and facilitate predictions. The taxon sampling is the most extensive to date and includes representatives of all five dryophthorine tribes and all but one subtribe. The phylogeny is based on secondary structural alignment of 18S and 28S rRNA totaling 3,764 nucleotides analyzed under Bayesian and maximum likelihood inference. We used a fossil‐calibrated relaxed clock model with two approaches, node‐dating and fossilized birth‐death models, to estimate divergence times for the subfamily. All tribes except the species‐rich Rhynchophorini were found to be monophyletic, but higher support is required to ascertain the paraphyly of Rhynchophorini with more confidence. *Nephius* is closely related to Dryophthorini and Stromboscerini, and there is strong evidence for paraphyly of Sphenophorina. We find a large gap between the divergence of Dryophthorinae from their sister group Platypodinae in the Jurassic‐Cretaceous boundary and the diversification of extant species in the Cenozoic, highlighting the role of coevolution with angiosperms in this group.

## Graphical abstract

This study presents the first phylogeny of the palm weevil subfamily Dryophthorinae (1,200 species, 153 genera), a group that is mainly associated with monocots and includes major pests, such as the Red and South American Palm Weevils. This study includes genetic sampling of all major lineages worldwide, integrated structural alignment of ribosomal DNA and cutting‐edge fossil‐calibrated dating analyses. Major findings include confirmation of an unstable classification system; weak and unreliable diagnostic characters, and diversification coinciding with the dominance of flowering plants. The phylogeny enables pest management personnel and researchers to not only make solid predictions about potential new pests, unknown host associations, potential vectors of invasives, endosymbionts, and natural enemies, but also provides insights into the complicated process of diversification of phytophagous insects.

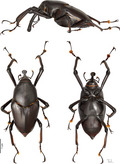

## 1 INTRODUCTION

The morphologically distinctive weevil subfamily Dryophthorinae is among the most economically important and culturally embedded insect groups known (Rugman‐Jones et al., 2013; van Huis et al., 2013). It includes enormous palm weevils, tiny rice weevils and many other species that attack tropical and temperate crops, from palms, pineapple, bamboo, and banana to sugarcane, corn, wheat and rice. Dryophthorine weevils occur on every continent, in rainforests, deserts and grasslands, they have a deep fossil record and are prominently known from ancient grain stores in Egyptian pyramid and Pleistocene caves (Panagiotakopulu, 2001; Panagiotakopulu & Buckland, 2017). Dryophthorines are also models for study of weevil development (Davis, 2011) and microbial endosymbiosis (Heddi & Nardon, 2005; Lefevre et al., 2004). Because of their widespread economic importance, the first weevil (after the forest pest scolytine *Dendroctonus ponderosae*) to have a genome published was the dryophthorine red palm weevil (Hazzouri et al., 2020). It is therefore surprising that there has never been a broadly sampled molecular phylogenetic study of this significant, and otherwise extraordinarily well‐known, weevil subfamily.

The Dryophthorinae are ecologically distinctive, and among the few insect higher taxa to specialize almost entirely on monocots. The vast majority of the 153 genera of Dryophthorinae, especially the species‐rich Rhynchophorini, are associated with stressed, dying or dead monocotyledonous angiosperms (Anderson & Marvaldi, 2014; Oberprieler et al., 2007). Due to the close association with monocots and their preference for plants that may be stressed by conditions sometimes present in agricultural settings such as drought, monoculture, or disease, dryophthorines include a number of serious pest of economically important plants (Anderson & Marvaldi, 2014; Oberprieler et al., 2007; Zimmerman, 1968a, 1968b, 1993). Members of Dryophthorini, Stromboscerini and Orthognathini are saprotrophic, feeding on decomposing seed plants (flowering plants, conifers, etc) (Anderson & Marvaldi, 2014; Gardner, 1934, 1938; Vaurie, 1970a, 1970b, 1971) with the majority of adult Dryophthorini and Stromboscerini residing in the leaf litter or decaying wood (Gardner, 1934, 1938; Grebennikov, 2018a, 2018b, 2018c). Perhaps the most economically important and widespread weevil genus is *Sitophilus* Schoenherr (subtribe Litosomina), which are major pests of stored grains (Plarre, 2010) everywhere that grains are harvested and have been for thousands of years (Panagiotakopulu, 2001; Panagiotakopulu & Buckland, 2017). Observing vast numbers of these weevils in grain shipping areas in Australia, Zimmerman (1993) proposed that they may also be among the most abundant insects on earth. Interestingly, *Sitophilus linearis* (Herbst) (Cotton, 1920) and *Tryphetus incarnatus* (Gyllenhaal) are departures from monocot grain‐feeding and develop instead within fabaceous seed pods [pers. obs.]. Other departures from monocot feeding are the genus *Phacecorynes* Schoenherr, known to be associated with dead or dying cycads (Tang & Oberprieler, 2006; Tang et al., 1999) and the Cryptodermatini, recently observed to prefer Marattiaceae (ferns [pers. obs]); both cases signify striking host‐plant shifts within the Dryophthorinae. In addition to varied diet preferences, the subfamily also exhibits extreme size variation from 1.5 mm to more than 28 mm. The saprotrophic Dryophthorini and Stromboscerini rarely reach more than 4 mm in length. The exception being the much larger *Nephius* Pascoe (6 mm) and *Stromboscerus* Schoenherr (6 mm) in Stromboscerini and a number of uncharacteristically large (5 mm or more) *Dryophthorus* Germar species that radiated on the Hawaiian archipelago. Dryophthorini and Stromboscerini have been previously classified in or near Cossoninae (Morimoto, 1962), which are also small and feed on dead or decaying organic matter. On the other hand, Rhynchophorini includes some of the largest known beetles, including the East Asian *Mahakamia* Ritsema that measures more than 25 mm excluding the elongate legs (with legs 70 mm), or *Protocerius colossus* (Olivier) measuring more than 28 mm. This extreme size variation within a single subfamily is rare in the natural world and studying the evolution of this group may provide insights into what is driving this phenomenon.

Dryophthorinae includes an estimated 1,200 species in 153 extant genera and five tribes: Cryptodermatini; Dryophthorini; Stromboscerini; Orthognathini; and Rhynchophorini (Alonso‐Zarazaga & Lyal, 1999; Anderson & Marvaldi, 2014; Oberprieler et al., 2007). Marvaldi and Morrone (2000) recognized the monophyly of the subfamily and eight tribes following the classification by Kuschel (1995): Cryptodermatini, Dryophthorini, Orthognathini, Rhinostomini, Rhynchophorini, Sitophilini, Sphenophorini, and Stromboscerini; but this classification was later slightly modified by Alonso‐Zarazaga and Lyal (1999). At the time, the group was treated as a family within the Curculionoidea, but their subfamily status is now widely accepted (Chamorro, 2019; Marvaldi et al., 2009; Oberprieler et al., 2007). It is among the few Curculionidae subfamilies for which natural limits are well understood (Marvaldi et al., 2002, 2009; Mugu et al., 2018; Shin et al., 2017). Historically, species in the tribes Dryophthorini and Stromboscerini had been classified with weevils in the subfamily Cossoninae, but evidence for monophyly of Dryophthrorinae as currently classified has gained support through the study of the immature stages (Anderson, 1948; Marvaldi, 1997; May, 1993, 1994), adults (Kuschel, 1995; Thompson, 1992; Zimmerman, 1993), and molecular data (Gunter et al., 2016; Haran et al., 2013; Marvaldi et al., 2009; McKenna et al., 2009; Mugu et al., 2018; Shin et al., 2017). Of these studies, McKenna et al. (2009) included nine representatives of the subfamily to understand the higher‐level relationships of Curculionoidea and recovered a monophyletic Dryophthorinae without Stromboscerini. The 18S and 28S Bayesian analysis by Marvaldi et al. (2009) recovered Stromboscerini (represented by a single exemplar) as the sister group to bagoine brachycerines, while the remainder of the dryophthorines were recovered sister to Platypodinae. However, monophyly was confirmed in the combined molecular and morphological parsimony analysis of that same study (Marvaldi et al., 2009). Other studies (Gunter et al., 2016; Haran et al., 2013; Shin et al., 2017) recovered the family as monophyletic, yet the taxon sampling was not as extensive as in Marvaldi et al. (2009) and McKenna et al. (2009).

Relationships within the subfamily, however, remain untested with three notable exceptions: Morrone and Cuevas (2009), Brian O'Meara's unpublished Harvard University honors thesis (O'Meara, 2001) and Grebennikov (2018a, 2018b, 2018c). Morrone and Cuevas (2009) performed a morphology‐based analysis of the genera now included in Orthognathini. Their study also included 10 outgroup taxa, including eight dryophthorines representing four tribes and subtribes (Stromboscerini, Dryophthorini, Rhynchophorini (Litosomina, Sphenophorina, Rhynchophorina)). The authors recovered *Rhinostomus* Rafinesque subtending the remaining Orthognathini. Based on their results, they recognized a single tribe Orthognathini with two subtribes, Rhinostomina and Orthognathina. The monophyly of the subfamily was well‐supported and, while not the central goal of the study, the smaller‐sized, decomposing wood‐associated dryophthorines (Stromboscerini and Dryophthorini) were found to be monophyletic and closer to the root of the tree as the sister group to the rest of the dryophthorines. O'Meara's thesis (2001) remains the most complete study of the subfamily to date with 26 ingroup taxa consisting of members of Orthognathini and Rhynchophorini (Litosomina, Rhynchophorina, Sphenophorina, and Diocalandrina) and based on 3 molecular markers (COI, EF‐1a, 28S). The study did not, however, include Stromboscerini and Dryophthorini, groups previously included in Cossoninae. Relationships at the base of the tree were mostly unresolved, with paraphyletic Orthognathini and Sphenophorina, *Cosmopolites* Chevrolat(Sphenophorina) sister to Rhynchophorina and *Sitophilus* (Litosomina) sister to Diocalandrina (O'Meara, 2001). Grebennikov (2018a, 2018b, 2018c) made considerable advancement toward our understanding of Stromboscerini in a series of studies, including molecular‐based phylogenetic analyses of several members of the tribe based on three molecular markers (COI, ITS2, 28S) (Grebennikov, 2018a, 2018c). The genus *Nephius* was recovered as sister to a monophyletic Dryophthorini + Stromboscerini (*Allaeotes* Pascoe, *Dexipeus* Pascoe, *Orthosinus* Motschulsky, *Tasactes* Faust, *Tetrasynommatus* Morimoto) and the author suggested the possible exclusion of *Nephius* from Stromboscerini. Relationships among genera of the most diverse and economically important tribe Rhynchophorini, of the poorly studied Oriental tribe Cryptodermatini and the monotypic Polytina and Diocalandrina also remain untested.

The age of Dryophtorinae is currently poorly known. O'Meara (2001) estimated the group to have originated approximately 70 million years ago by using a fixed clock rate of the COI gene. In McKenna et al. (2009), the nine representatives of the subfamily diverged during the mid to late‐Cretaceous period (65–115 myo). In both cases, dates are derived from phylogenies with small sampling of extant species and even less of the relatively rich fossil record for the subfamily. Therefore, there is still a poor understanding regarding the timing of dryophthorine evolution and how this group achieved a pantropical distribution.

The major goal of this study is to infer a phylogeny of Dryophthorinae to not only provide insights into the process of diversification of phytophagous insects, but also to inform classification, facilitate predictions and improve our understanding of potential pests with unknown host associations, natural enemies, and endosymbionts. Here we infer, for the first time, a comprehensive molecular phylogeny of Dryophthorinae including representatives of all tribes and all but one subtribe, and use a relaxed clock model calibrated with fossils to infer the timing of their diversification.

## 2 MATERIALS AND METHODS

### 2.1 Taxon sampling

The analysis includes a broad sampling of 62 ingroup taxa representing all five dryophthorine tribes and all but one subtribe, Ommatolampina, and a putative rhynchophorine African lineage that includes *Ichthyopisthen* Aurivillius, *Korotayeavius* Alonso‐Zarazaga and Lyal, among others. The outgroup consisted of two platypodine species in the genus *Euplatypus* Wood following recent phylogenetic studies suggesting a sister group relationship between Platypodinae and Dryophthorinae (Gillett et al., 2014; Haran et al., 2013; Marvaldi, 1997; McKenna et al., 2009; Mugu et al., 2018; Shin et al., 2017). Taxon sampling is a mixture of trusted GenBank sequences (Appendix S2) and newly generated sequence data now available in GenBank (Appendix S3). Voucher specimens are deposited at the National Museum of Natural History (USNM) (Appendix S3).

### 2.2 PCR protocols

DNA was extracted from one or more legs per specimen using the Autogen Prep 965 phenol‐chloroform automated extractor (Autogen). The sample of each specimen was digested overnight at 55°C in a proteinase‐k buffer in a shaking incubator and then extracted on the Autogen using the manufacturer's animal tissue protocol. Extracted DNAs were resuspended in 50 µl solution.

The small ribosomal subunit (18S) was amplified with primers 18SA/18SB (Medlin et al., 1988) which was followed by reamplification using internal primers 18SL, 18SC, 18SY, 18SO (Apakupakal et al., 1999). Sequencing used all original and internal amplification primers. The large ribosomal subunit (28S) was amplified and sequenced with primers LS58F/LS998R and NLF184‐21/LS1041R (Maddison, 2012). The 10 µl PCR mix contained 0.3 µl of each 10 µM primer, 0.5 µl dNTPs (2.5 mM each), 0.1 µl GoTaq Hot Start Master Mix (Promega), and 0.1 µl 20 µg/µl BSA. The reaction mix for 28S additionally contained 0.1 µl DMSO. The PCR temperature profile for all sets of primers consisted of an initial denaturation at 95°C (5 min), followed by 35 cycles of denaturation at 95°C (30 s), annealing at 48–52°C (30 s) and extension at 72°C (45 s) followed by a final extension at 72°C (5 min). The majority of the reactions used an annealing temperature of 50°C. For cycle sequencing 30 cycles of 95°C (30 s), 48°C (30 s) and 60°C (4 min) were employed.

### 2.3 Alignment

Two molecular markers, 18S and 28S rRNA were aligned using primary and secondary structure, totaling 3,764 base‐pairs. Alignment was implemented in Geneious 9.1 (https://www.geneious.com) following rRNA secondary structure models for phylogenetic reconstructions published in Marvaldi et al. (2009). The GenBank 18S and 28S sequences of *Rhynchophorus palmarum* (Linnaeus) uploaded by Marvaldi et al. (2009) were used as reference for an initial automated alignment in Geneious. The annotation feature in Geneious was used to verify and subsequently align by eye the various stems and loops across all taxa for both loci. To test the effect of using secondary structure for alignment, we additionally used alignments produced with MUSCLE (Edgar, 2004), without secondary structure information, as input data for phylogenetic analyses. The data were concatenated, taxa renamed, and exported in NEXUS format including MrBayes commands in Mesquite v. 3.61 (Maddison & Maddison, 2019).

### 2.4 Uncalibrated trees

We inferred the phylogenetic tree of Dryophthorinae under maximum likelihood with IQTREE version 2.0.3 (Minh et al., 2020). For the alignments using secondary structure, we used annotations for rRNA loops and stems as 13 starting partitions in ModelFinder implemented in IQTREE (Kalyaanamoorthy et al., 2017) with default parameters to select the best partitioning scheme and DNA substitution models, allowing all time‐reversible substitution models allowed by IQTREE. The best partitioning scheme, with nine partitions (Appendix S4), was used in the search for the best tree (Chernomor et al., 2016), with support evaluated with ultrafast bootstrapping (Hoang et al., 2018). We also obtained trees under Bayesian inference with MrBayes v.3.2.7a (Huelsenbeck & Ronquist, 2001; Ronquist & Huelsenbeck, 2003). As with the maximum likelihood analysis, we started from 13 partitions defined by rRNA stem and loops, and used PartitionFinder 2 (Lanfear et al., 2017) with phyML (Guindon et al., 2010) and a greedy algorithm (Lanfear et al., 2012) to select the best partition scheme under the Bayesian Information Criteria, constraining DNA substitution models to those implemented in MrBayes. The nine partitions identified by PartitionFinder and their respective substitution models (Appendix S4) were used in all Bayesian analyses. We ran 2 MCMC chains with 4 Metropolis‐coupled runs for each for 20 million generations sampled every 1,000 generations. The first 25% of the samples were discarded as burn‐in, and convergence was checked by the average standard deviation of split frequencies as reported by MrBayes and by Effect Sample Sizes for all parameters in Tracer version 1.7.0 (Rambaut et al., 2018). The posterior distribution of trees was summarized by the majority‐rule consensus. Both Bayesian and Maximum likelihood analyses were repeated with alignments without secondary structure information, but in this case we only included two initial partitions for IQTREE or PartitionFinder, with the respective partitioning schemes (Appendix S4) used as input in tree searches. Trees were visualized with FigTree version 1.4.4 (Rambaut & Drummond, 2018), rooted with the outgroup, and exported image files were further edited for final figure preparation.

### 2.5 Divergence time estimates

We used a relaxed clock model calibrated with fossil occurrences to estimate divergence times for Dryophthorinae in MrBayes 3.2.7a with two approaches: node‐dating and the fossilized birth‐death (FBD) model (Heath et al., 2014; Stadler, 2010; Zhang, 2016). We compiled a list of fossil occurrences for Dryophthorinae from the literature (Table 1), with age of fossil localities based on Legalov (2020) for the Paleogene. More recent fossils include Dominican amber with an age range of 15–20 My (Penney, 2010) and the upper Miocene of Cantal with 6.5–10 My (Gibert et al., 1977).

**TABLE 1:** Fossil data used for calibration

Taxon species	Reference	Taxon	Age
*Rhinoporkus gratiosus* Legalov, Kirejtshuk, Nel	Legalov et al. (2019)	Dryophthorini	Early Eocene, Oise amber (55.8–48.6)
*Stenommatus leptorhinus* Poinar & Legalov	Poinar and Legalov (2014)	Dryophthorini	Oligocene to Miocene, Dominican amber (15–20 My)
*Stenommatus pulvereus* Davis & Engel	Davis and Engel (2006)	Dryophthorini	Oligocene to Miocene, Dominican amber (15–20 My)
*Stenommatus tanyrhinus* Poinar & Legalov	Poinar and Legalov (2014)	Dryophthorini	Oligocene to Miocene, Dominican amber (15–20 My)
*Dryophthorus acarophilus* Davis & Engel	Davis and Engel (2007)	*Dryophthorus*	Oligocene to Miocene, Dominican amber (15–20 My)
*Dryophthorus microtremus* Poinar & Legalov	Poinar and Legalov (2014)	*Dryophthorus*	Oligocene to Miocene, Dominican amber (15–20 My)
*Dryophthorus superbus* (Pitons)	Zherikhin (2000)	*Dryophthorus*	Late Miocene to Early Pliocene (5–7 My)
*Sitophilus punctatissimus* Zherikhin	Zherikhin (2000)	Litosomina	Late Miocene (7–10 My)
*Mesocordylus longiscapus* Davis & Engel	Davis and Engel (2009)	*Mesocordylus*	Oligocene to Miocene, Dominican amber (15–20 My)
"grosser Rüsselkäfer" Orthognathini	Schlee (1990): fig 50	Orthognathini	Late Eocene, Baltic amber (33–48 My)
*Bicalcasura maculata* Poinar & Legalov	Poinar and Legalov (2014)	Rhinchophorini	Oligocene to Miocene, Dominican amber (15–20 My)
*Oryctorhinus tenuirostris* Scudder	Scudder (1893)	Sphenophorina	Early Oligocene, Florissant (33–35 My)
*Sphenophorus. elegans* Théobald, 1935	Piton & Thèobald 1935	Sphenophorina	Late Miocene, Cantal (6–10 My)
*Scyphophorus fossionis* Scudder, 1891	Scudder (1893)	Sphenophorina	Early Oligocene, Florissant (33–35 My)
*Scyphophorus laevis* Scudder, 1891	Scudder (1893)	Sphenophorina	Early Oligocene, Florissant (33–35 My)
*Sphenophorus naegelianus* Heer, 1847	Scudder (1891)	Sphenophorina	Late Miocene, Oeningen (5–7 My)
*Sphenophorus proluviosus* von Heyden & von Heyden, 1866	Scudder (1891)	Sphenophorina	Late Oligocene, Rott (23–24 My)
*Sphenophorus. regelianus* Heer, 1847	Scudder (1891)	Sphenophorina	Late Miocene, Oeningen (5–7 My)
*Scyphophorus tertiarius* Wickham, 1911	Scudder (1895)	Sphenophorina	Early Oligocene, Florissant (33–35 My)
*Palaeodexipeus kirejtshuki* Legalov	Legalov (2016)	Stromboscerini	Late Eocene, Baltic amber Kaliningrad (33–48 My)
Prob. *Orthosinus/Xerodermus*	Unpublished Chamorro	Stromboscerini	Late Eocene, Baltic amber Kaliningrad (33–48 My)
*Rovnoslonik damzeni* Legalov, Nazarenko, Perkovsky	Legalov et al. (2019)	Stromboscerini	Late Eocene, Rovno amber (33–48 My)
*Stenommatomorphus hexarthrus* Nazarenko	Nazarenko and Perkovsky (2009)	Stromboscerini	Late Eocene, Rovno amber (33–48 My)

For node dating, we considered only the oldest fossil occurrence of each higher taxon, when more than one species was sampled for the phylogeny. In each case, we constrained crown groups to be monophyletic and assigned offset lognormal priors to their ages. The age of crown group Dryophthorinae was assigned an offset of 48.6 My, mean of 60.8 My and standard deviation of 1.5 My, following the oldest fossil *Rhinoporkus gratiosus* Legalov et al. (2019) in Oise amber. To the crown group Dryophthorini we assigned a prior with offset of 15 My, mean of 25 My and standard deviation of 1.5 My, with a high probability in the interval of Dominican amber, in which the oldest fossils of *Dryophthorus* are found. The oldest occurrence of Sphenophorina, in the Florissant formation, were assigned to the most recent common ancestor of Sphenophorina and Rhynchophorina, excluding *Scyphophorus* Schoenherr + *Trigonotarsus* Guerin‐Meneville. We based this decision on the topology of the uncalibrated trees, which showed that Rhynchophorina is nested within the main group of Sphenophorina. We assigned to this node a prior distribution with offset of 33 My, mean of 40 My and standard deviation of 1.5 My. The two species of Platypodinae were used as outgroups, and to the root of the tree we assigned a prior truncated Normal distribution, with minimum of 48.6 My, mean of 139.4 My and standard deviation of 7.45 My. This follows the estimated split between Platypodinae and Dryophthorinae in Toussaint et al. (2017), a reanalysis of the dataset of McKenna et al. (2015). We used a birth‐death tree prior, with an Exponential prior distribution with mean 0.04 for the birth rate (Condamine et al., 2016) and a uniform prior for the death rate. The clock model was independent gamma rates (IGR) with an Exponential (mean = 1e‐2) prior distribution for the mean and Exponential (mean = 2e‐4) prior distribution for variance. This analysis was done using the alignment with secondary structure and same partitioning scheme as in the uncalibrated tree. We ran the MCMC for a total of 10 million generations, with other run settings, burn‐in and chain mixing evaluation being the same as the uncalibrated tree in MrBayes.

For the FBD model, we included all fossils in Table 1, assigning their possible time range as uniform priors. The position of each fossil was constrained to the appropriate higher taxon by using topology constraints. In most cases, these were hard monophyly constraints, but a different approach was needed for fossils identified as Sphenophorina, which resulted not to be monophyletic in the uncalibrated analyses. In this case, we used partial constraints to allow fossils to be assigned to any of the three clades of Sphenophorina, and to the stem nodes of Sphenophorina(part) + Rhynchophorina but not to Rhynchophorina itself. A similar approach was used for *Bicalcasura maculata*, allowed to float between the different clades of Rhynchophorini. The only node to which we assigned a prior is the split between Platypodinae and Dryophthorinae, which received the same prior as in Node‐dating analysis. We used the fossilized birth‐death process as tree prior, constraining fossils to be tips, not ancestors. Birth rate, death rate and clock priors were specified as for the node‐dating analysis, and we used a fossilization rate prior distribution Beta (1,10) to indicate that the fossilization rate for insects should be small. The MCMC run parameters were the same as node calibration. Prior to tree summarization, we removed fossil tips using scripts from Kim et al. (2018).

Since uncalibrated trees revealed an unresolved polytomy at the base of Dryophthorinae, and some of the higher taxa had low support, we implemented topological constraints in both calibrated analyses following the topology of the unconstrained Bayesian tree based on structural alignment. These included monophyly constraints for the 3 separate groups of Sphenophorina, and also for Litosomina, Rhynchophorina, Dryophthorini, Stromboscerini, Rhinostomina, *Dryophthorus*, *Mesocordylus*, *Stenommatus*, Litosomina + Polytina + Diocalandrina + Cryptodermatini, and Orthognathini + Stromboscerini + Dryophthorini. Other than these constraints, the position of the clades in the unresolved basal polytomy in Dryophthorinae was free. Both calibration analyses were summarized by their maximum clade credibility trees with SumTrees (Sukumaran & Holder, 2010, 2015) and plotted with ggtree version 2.2.1 (Yu, 2020; Yu et al., 2017).

## 3 RESULTS

**FIGURE 1:**
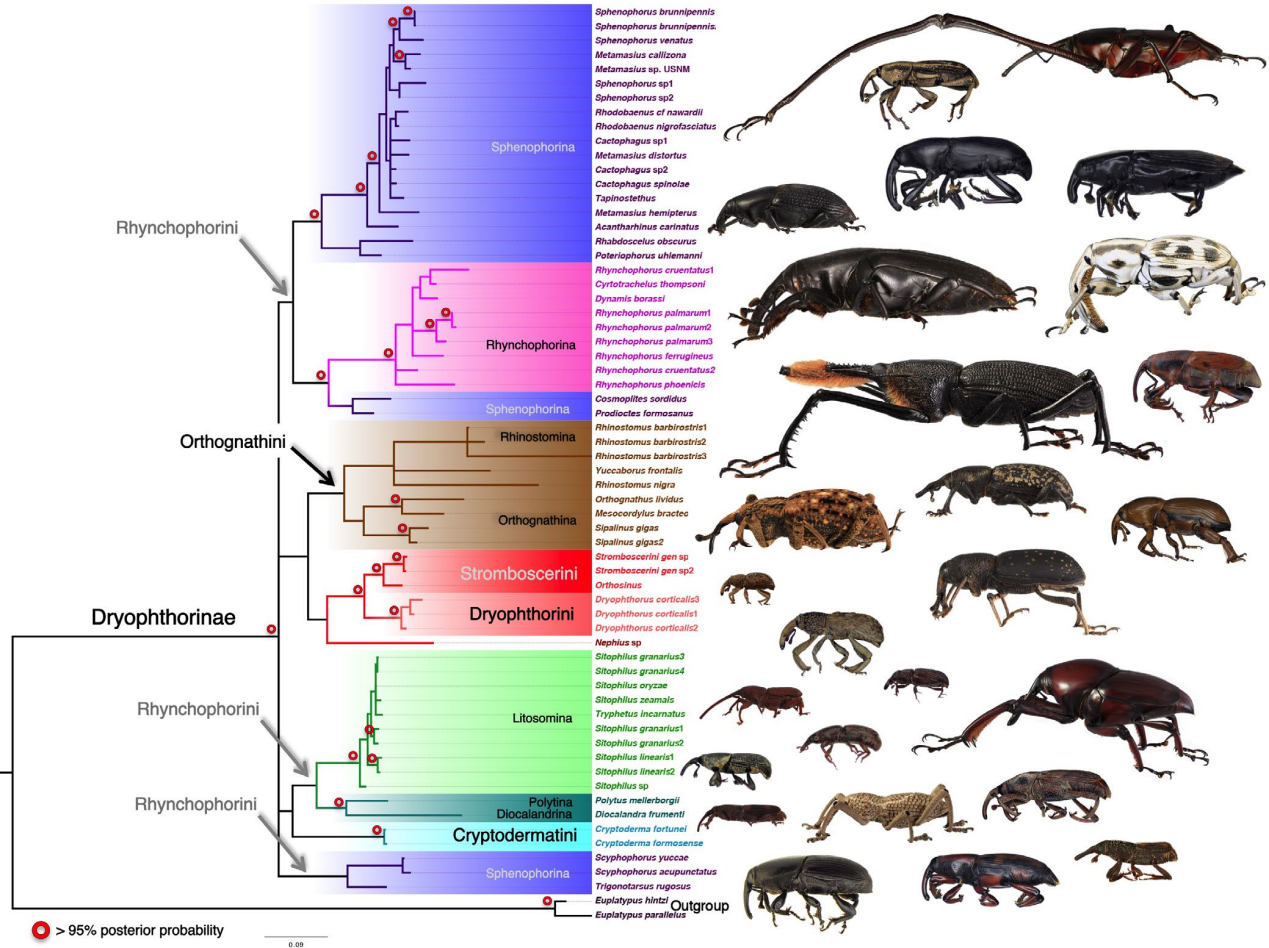
Bayesian topology with posterior probabilities (denoted by open red circle) of Dryophthorinae of the combined 18S and 28S aligned using primary and secondary structure. The analysis includes a broad sampling of 64 taxa, 2 platypodine outgroup taxa and 62 ingroup taxa representing all 5 tribes dryophthorine tribes and all but 1 subtribe Ommatolampina and a putative African lineage. Lateral views of Dryophthorinae genera (From left to right and top to bottom: *Mahakamia* (long fore legs*); Rhabdoscelus; Acantharhinus; Cactophagus: Tapinostethus; Dynamis; Poteriophorus; Rhinostomus; Prodioctes; Sipalinus; Yuccaborus; Orthognathus; Stromboscerini; Nephius; Mesocordylus; Tryphetus; Dryophthorus; Cyrtotrachelus* (long fore legs)*; Polytus; Sitophilus; Diocalandra; Cryptoderma; Metamasius (=Paramasius); Scyphophorus; Metamasius; Myocalandra*

The Bayesian (Figure 1) and Maximum Likelihood (ML) (Appendix S1) analyses of 18S and 28S rRNA aligned using primary and secondary structures conflicted minimally. Both inference methods recovered strongly supported monophyletic Dryophthorinae (PP: 1.0; 100); Cryptodermatini, based on a single genus, (PP: 1.00; 100), Dryophthorini, represented by a single genus in this study, currently includes only three genera, (PP: 1.0; 100); Litosomina (PP: 1.0; 99); Rhynchophorina (PP: 1.00; 100) and Stromboscerini (PP: 0.99; 98) without *Nephius*. The anomalous stromboscerine *Nephius* was moderately supported (PP: 0.90; 96) as the sister taxon to a strongly supported monophyletic Stromboscerini + Dryophthorini (PP: 0.99; 99). In addition to a well‐supported monophyletic Litosomina, both topologies recovered with high support (PP: 96; 99), a sister relationship between the monotypic rhynchophorine subtribes Polytina + Diocalandrina and this clade sister to the remainder of the litosomines (PP: 0.96; 94) with high to moderate support. Another relationship with strong to moderate support (PP: 0.99; 89) and previously not hypothesized was Rhynchophorina sister to *Cosmopolites* and *Prodioctes* Pascoe. This placement of *Cosmopolites* and *Prodioctes* sister to rhynchophorines and the recovery of *Scyphophorus* and *Trigonotarsus* outside a strongly supported sphenophorine core (PP: 0.97; 90) renders the subtribe Sphenophorina paraphyletic or polyphyletic. It was the placement of *Trigonotarsus* as well as deeper splits that resulted in conflict among the two topologies. In the Bayesian analysis, *Trigonotarsus* was recovered as the sister taxon to a monophyletic *Scyphophorus*, while in the maximum likelihood analysis *Trigonotarsus* subtended the polytine + diocalandrine + litosomine clade, however, both relationships had weak support (PP: 0.64; 65). A monophyletic Orthognathini split into Orthognathina and Rhinostomina, as identified by Morrone and Cuevas (2009), was moderately supported (PP: 0.88; 86). With the exception of a well‐supported monophyletic *Mesocordylus* and *Orthognathus* (PP: 0.99; 100), relationships within orthognathines were moderately supported; however Bayesian and ML topologies did not conflict. *Cryptoderma*, the monotypic genus of the tribe Cryptodermatini, is recovered with weak support sister to the litosomine, Polytina + Diocalandrina clade in the Bayesian analysis or as sister to orthognathines, stromboscerines, and dryophthorines in the ML analysis. Both analyses recover with moderate support (PP: 0.71; 86) a clade containing orthognathines and stromboscerines + dryophthorines. Rhynchophorini was not recovered as monophyletic in either analysis.

Lower‐level relationships reveal a number of cases of genera and species recovered as nonmonophyletic. *Rhynchophorus* is paraphyletic with respect to *Cyrtotrachelus* and *Dynamis*. A sister group relationship between the latter two and *Rhynchophorus cruentatus* had bootstrap support of 96 and posterior probability of 0.61. *Sphenophorus*, *Metamasius*, *Cactophagus* were recovered as part of a core clade of Sphenophorina, but none of the three genera as monophyletic. *Sitophilus* was recovered as paraphyletic with respect to *Tryphetus*, but with low support. The paraphyly of *Sitophilus granarius* with respect to other species in the genus was also recovered with low support.

Bayesian (Appendix S5) and ML trees (Appendix S6) using the MUSCLE alignment did not substantially differ from the topologies based on secondary structural alignment, except for basal splits with very low support. The major differences were more strongly supported (PP: 0.95; 93) sister group relationships between *Nephius* and Stromboscerini + Dryophthorini; the lack of strong support for the sister relationship between Polytina + Diocalandrina or as the sister clade to the remainder of the litosomines (0.58; 79), instead *Tryphetus* was placed, with moderate to high support (0.74; 95), as sister to *Polytus*; the unresolved placement of Cryptodermatini; a monophyletic *Trigonotarsus* + *Scyphophorus* in a basal polytomy in the Bayesian tree but not in the maximum likelihood tree; and the clade containing orthognathines and stromboscerines + dryophthorines was better supported in the Bayesian analysis but not under maximum likelihood (0.91; 74).

**FIGURE 2:**
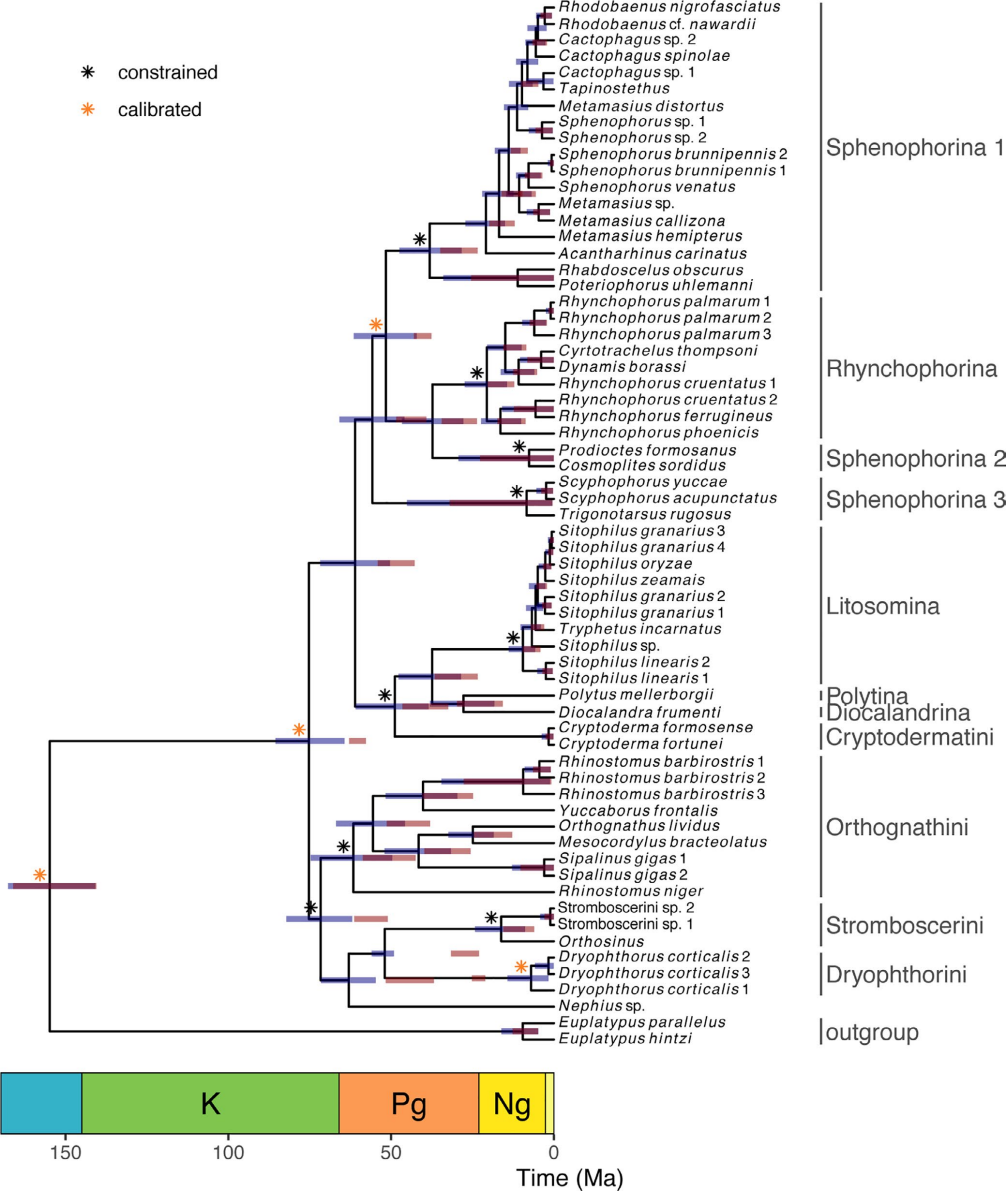
Summary of calibrated trees, with MCC topology obtained by the FBD model. Asterisks show nodes constrained to be monophyletic, with yellow asterisks further indicating nodes used as calibration in the node‐dating analysis. Error bars: blue = Fossilized birth‐death (FBD) model, red = Node dating

Intervals for node‐dating were smaller and more recent than those of FBD, but both overlap for most nodes (Figure 2). Major differences are the dates of Dryophthorini and Stromboscerini + Dryophthorini, which were found to be much more recent with node dating. The basal polytomy for Dryophthorinae found in uncalibrated analyses was resolved, probably as an emerging result of the topological constraints applied. In particular, *Scyphophorus* + *Trigonotarsus* was recovered as sister to the remaining Sphenophorina + Rhynchophorina. While the split of Dryophthorinae from its closest living relatives in Platypodinae dates back to the Jurassic‐Cretaceous boundary, the age of the crown group Dryophthorinae is inferred to about 100 million years later, at the Cretaceous‐Paleogene boundary in the case of the FBD model and in the early Paleogene in the case of node dating.

## 4 DISCUSSION

### 4.1 Systematics, morphology, and classification

#### 4.1.1 Rhynchophorini and included subtribes

Rhynchophorini (sensu Alonso‐Zarazaga and Lyal (1999)) was not recovered as a well‐supported monophyletic group in either of the uncalibrated analyses. In the Bayesian majority‐rule consensus tree, it forms three of the four groups in a basal polytomy of Dryophthorinae, and in the maximum likelihood tree *Scyphophorus* was found, with very low support, as closely related to a clade including all other tribes. Considering support values, these results are not incompatible with a monophyletic Rhynchophorini but show the need for additional data to resolve this question. There are only a handful of morphological characters currently known to support this taxon. These include an exposed pygidium beyond the elytral apex (Voss, 1958), as well as separated procoxae (Kuschel, 1995). Only the former appears to be consistently found in members of the Rhynchophorini.

The paraphyly of Sphenophorina with respect to Rhynchophorina is much more conclusive based on our results. The sister group relationship between Rhynchophorina and *Prodioctes* + *Cosmopolites* is well‐supported, and the placement of *Scyphophorus* and *Trigonotarsus* is uncertain. *Cosmopolites* has been previously considered as not part of Sphenophorina, but its placement was thought to be in the Litosomina, not Rhynchophorina (Anderson, 2002, 2003, 2018). The relationship between *Prodioctes* + *Cosmopolites* and Rhynchophorina has never been proposed before. The creation of new subtribes or the transfer of sphenophorine genera to rhynchophorina may be warranted in the future.

**FIGURE 3:**
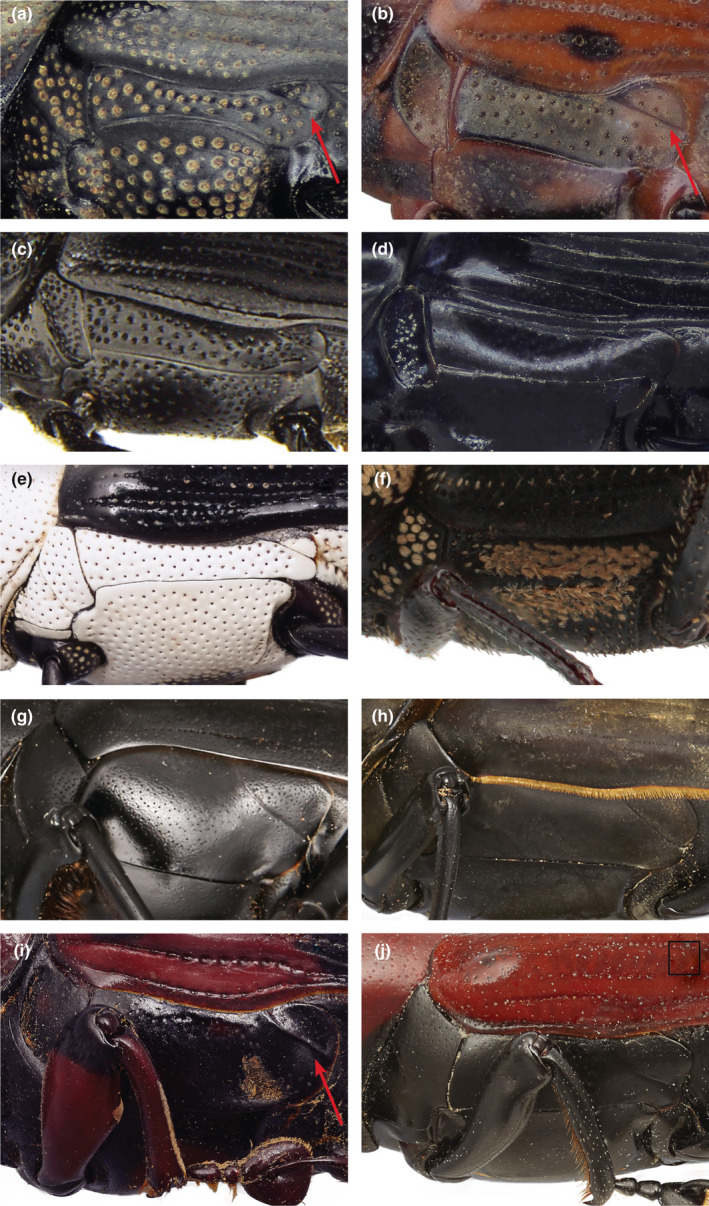
Detail of Metanepisternum and Metepimeron. Arrows show sulcus separating the metanepisternum and metepimeron. (a) *Cosmopolites sordidus*; (b) *Prodioctes*; (c) *Scyphophorus acupunctatus*; (d) *Tapinostethus*; (e) *Cercidocerus*; (f) *Eucalandra*; (g) *Dynamis*; (h) *Macrocheirus*; (i) *Tetratopos*; (j) *Aphiocephalus*

The shape and size of the thoracic metepimeron and metanepisternum have played a key role in distinguishing Rhynchophorini subtribes Sphenophorina and Rhynchophorina (Kuschel, 1995; Voss, 1958). Rhynchophorina have a broader more parallel‐sided metanepisternum whereas Sphenophorina have a metanepisternum that narrows caudally. Examination of the metanepisternum and metepimeron present in sphenpohorine and rhynchophorine genera (Figure 3), including three of the four genera not recovered within core sphenophorines, suggests a rather unique type of horizontal sulcus separating the metanepisternum and metepimeron in *Cosmopolites* and *Prodioctes*, which differs from core Sphenophorina (Figure 3a, b; arrow). This character appears to also be present in at least one other sphenophorine genus not included in this study, *Tetratopos* Chevrolat (Figure 3i).

The close relationship between *Cosmopolites* and *Prodioctes* is also supported by larval habits, including species with root or rhizome boring larvae (Jones, 1941). *Cosmopolites*, a genus containing two species, including the Banana Root Borer, *Cosmopolites sordidus* (Germar), has in the past been included in Litosomina (Anderson, 2002, 2003, 2018) or Sphenophorina (Alonso‐Zarazaga & Lyal, 1999) and *Prodioctes*, which includes the Cardamon or Rhizome weevil, *Prodioctes haematicus* Chevrolat, have always been included in Sphenophorina (=Calendrini; =Sphenocorynina) (Kuschel, 1995; Voss, 1958).

A type of leg interlocking device, formed by modification of the femoral‐tibial articulation, helps distinguish sphenophorines and rhynchophorines from litosomines (Kuschel, 1995). However, this character has at times been difficult to interpret (Anderson, 2018). Anderson (2018) found the femoral‐tibial articulation to be of at least two kinds: one where the interlocking brackets are formed by a swelling of the ventrally directed flanges at the distal end of the femora and the second where the ventrally directed flanges are instead inflexed, which is the typical type in Rhynchophorina and Sphenophorina. The first type was present in genera Anderson (2018) included in Litosomina (*Daisya* Anderson, *Melchus* Lacordaire, *Toxorhinus* Lacordaire and *Stockwellius*) and the second type was present in *Cosmopolites* and *Eucalandra* Faust (a genus with uncertain placement not included in this study). Anderson (2018) included *Cosmopolites* and *Eucalandra* and the newly described *Stockwellius* Anderson (2018) in Litosomina due to the shape of the scutellum and the presence of a premucro; however, until recently, *Cosmopolites* and *Eucalandra* were included in Sphenophorina (Alonso‐Zarazaga & Lyal, 1999), mainly hinging on the presence of this unique type of femoral‐tibial articulation (Kuschel, 1995). Nonetheless, Anderson (2018) retained both these genera in Litosomina pending further study. Our study suggests the placement of *Cosmopolites* needs to be reassessed and the femoral‐tibial articulation character examined for all rhynchophorines. An exhaustive comparative morphological study is currently underway with the aim of exploring character space to provide more reliable adult and larval characters (Chamorro, 2019) to circumscribe the groups.

*Scyphophorus* is a New World genus containing two species that specialize on the large, xeric‐adapted Asparagaceae *Agave* and *Yucca* in stressed, dead or dying condition. The larva of *Scyphophorus* is morphologically distinctive from most other sphenophorine larvae, but shares characteristics with Australasian *Trigonotarsus*, associated with *Xanthorrhoea* (Chamorro, 2019; May, 1994; Zimmerman, 1993), and the Afrotropical cycad‐specialist *Phacecorynes*. All of these prefer stressed, dead or dying host plants (Tang & Oberprieler, 2006; Tang et al., 1999), develop deep within the stem or trunk, and the larvae possess a pair of elongate caudal digitate projections on segment IX (Chamorro, 2019). Other genera with somewhat similar, but less pronounced, processes on segment IX, are *Rhodobaenus* and *Diathetes* (Chamorro, 2019). The function of these caudal processes on the larvae of these three genera is unknown. Zimmerman (1993) described the monotypic *Trigonotarsus* as singular and relictual without known close relatives. All three of these genera, *Scyphophorus*, *Trigonotarsus*, and *Phacecorynes*, occupy similar ecological niches, boring deep in above‐ground stems and trunks (Chamorro et al., 2016; Tang & Oberprieler, 2006; Tang et al., 1999; Zimmerman, 1993) of different lineages of putative morphologically convergent plants found on three separate geographic regions (North and South America; Australia; and southern Africa). In the Bayesian topology, *Scyphophorus* and *Trigonotarsus* were recovered with weak support together in a clade. In the ML topology *Trigonotarsus* was associated with the litosomines. Their estimated divergence, assuming monophyly, is highly uncertain and the 95% credibility interval covers most of the Neogene and Paleogene with both calibration models. This is a likely result of patterns of missing data in the alignment, with only 28S sequences available for *Scyphophorus* and only 18S for *Trigonotarsus*. *Phacecorynes* was not included in this study but specimens are now available for sequencing. The larval morphology and habitus, together with our results, are suggestive of the existence of a currently unrecognized lineage, possibly dating close to origin of extant Dryophthorinae, for which only one (*Trigonotarsus*), two (*Scyphophorus*), and three (*Phacecorynes*) species are known today.

Whether Ommatolampina and Rhynchophorina are monophyletic (Kuschel, 1995) remains to be determined since no representative of Ommatolampina was included in this study. Ommatolampines differ from other members of the tribe in usually having divaricate mandibles and a shorter metanepisternum (Voss, 1958).

Both analyses moderately support a close relationship between Polytina and Diocalandrina with Litosomina, which supports Morimoto (1978) and Kuschel's (1995) hypotheses of relationships. The monotypic genus *Polytus*, which includes the species *Polytus mellerborgii* (Boheman) or the small banana weevil, has been placed initially in Litosomina (=Sitophilini) (Kuschel, 1995; Morimoto, 1978), Sphenophorina (=Sphenophorini, (Zimmerman, 1968)) and currently in its own rhynchophorine subtribe Polytina (=Polytini), which was erected by Zimmerman (1993). Diocalandrina, a rhynchophorine subtribe also established by Zimmerman (1993), includes *Diocalandra*, a palm‐feeder (Zimmerman, 1993) and *Myocalandra*, a borer of bamboo and rattan (Beeson, 1941; Kalshoven, 1961; Zimmerman, 1993). Diocalandrines superficially resemble species of the mostly grain or seed associated species of *Sitophilus*. However, diocalandrines lack a dorsal longitudinal sulcus on the pygidium, males lack sternite IX (spiculum gastrale) and a straight‐loop rectal valve (Zimmerman, 1993). Whether these features are shared with other morphologically similar members currently included in Litosomina, remains to be determined.

#### 4.1.2 Cryptodermatini

The relationship of Cryptodermatini to the rest of the dryophthorines remains unresolved. Bayesian analyses place them sister to Litosomina, Polytina and Diocalandrina, the maximum likelihood topology favors a placement with Orthognathini + Dryophthorini. *Cryptoderma* possess putative nongeniculate antennae bearing 11 antennomeres and are brachypterous. The biology and morphology of the immature forms remain unknown.

#### 4.1.3 Placement of Nephius and the monophyly of Stromboscerini

Stromboscerini is recovered within Dryophthorinae, which supports findings by Anderson (1948), Chamorro (2019), and Shin et al. (2017). Within Stromboscerini, *Nephius* and *Stromboscerus*, both bearing ocular lobes on the anterior margin of the prothorax, were considered by Grebennikov (2018a, 2018c) and Morimoto (1985) to be “aberrant” stromboscerines and possibly unrelated to the remainder of the members of the tribe. A molecular‐based phylogenetic analysis of several members of the tribe based on three molecular markers (COI, ITS2, 28S) (Grebennikov, 2018a, 2018c), recovered *Nephius* as sister to a monophyletic Dryophthorini + Stromboscerini and the author suggested the possible exclusion of *Nephius* from the tribe. Our study does not contradict those findings. *Nephius* (=*Anius*) has been included in Sipalini (=Orthognathina) (Voss, 1940) and uncertainty remains whether *Nephius* represent one or two genera based mainly on the presence‐absence of elytral humeri. Anderson (1948) disputed the placement of *Nephius* (=*Anius*) in Sipalini based on the lack of key characters of the larva and concluded that *Nephius* (=*Anius*) was instead closely related to Stromboscerini. Anderson (1948) also included Dryophthorini and Stromboscerini collectively under the then subfamily Stromboscerinae. In a recent study of the immature forms of Dryophthorinae (Chamorro, 2019), the larvae of both *Sipalinus* and *Nephius* were found to share a number of key features, including the presence of digitate processes on segments VIII and IX. However, *Nephius* bears spiracles on segments VII and VIII, while *Sipalinus* appears not to have functional spiracles. Several other characters of the antenna and mouthparts are also not shared. Our study does not support a sister relationship between *Sipalinus* and *Nephius*. The placement of *Stromboscerus* also remains uncertain (Grebennikov, 2018a, 2018b). *Stromboscerus* has been extremely difficult to collect to include in molecular studies (Grebennikov, pers. comm.), but with new techniques allowing the use of museum specimens, we expect to be able to include this genus as well as many other rare dryophthorines in future studies.

#### 4.1.4 Nonmonophyletic genera and species

While lower‐level relationships are not the main goal of this study, the inclusion of multiple representatives for some genera revealed paraphyly in some of them. In most cases, these relationships had low support, but *Metamasius* and *Sphenophorus* were found to be paraphyletic with high support. The last thorough revisions of both genera were done more than 50 years ago (Vaurie, 1951, 1966, 1967) and the generic limits have never been tested with phylogenies, highlighting the need for modern revisions of these genera.

### 4.2 Timing of dryophthorine evolution

Divergence time estimates from both methods were largely in agreement, with the notable exception of Dryophthorini and the fact that node‐dating estimates were generally less uncertain and more recent. These results are similar to those obtained in a phylogeny of prionine longhorn beetles (Kim et al., 2018), and simulations show that the usage of monophyly constraints instead of morphological matrices may lead to slightly overestimated ages for FBD (Luo et al., 2020). The mismatch in the ages of Dryophthorini is likely a result of an excessively young prior to the crown age for this group, since we sampled only a few species. The Dominican amber fossils are likely all in the stem group in relation to the species of Dryophthorini sampled here, which can be better accommodated by the FBD model than by node dating. Given that otherwise both inferences are very similar but FBD uses the totality of fossil information instead of a few calibration points, we will consider mostly the FBD results here.

Divergence time estimation shows a large gap of about 100 million years between the divergence from platypodines and onset of diversification of extant dryophthorines. This contrasts with the patterns for platypodines, in which much of the diversification of the paraphyletic Tesserocerini took place in the Cretaceous (Jordal, 2015). The crown age of extant dryophthorines is inferred at the Cretaceous‐Paleogene transition, and no fossils are known from the Mesozoic. This large gap between the origin of the group in early Cretaceous and the diversification of extant species in the Cenozoic has also been observed in most angiosperm families, including monocots (Ramírez‐Barahona et al., 2020). It is therefore likely that dryophthorine diversification pattern is a result of their early association with angiosperms. Platypodines, on the other hand, are less specialized on their plant hosts due to their cultivation of fungi, and their diversification is more independent of the history of this plant group (Jordal, 2015). The relatively young age of the different groups of Dryophthorinae in the Cenozoic suggests that the pantropical distribution of the group has been achieved mostly by dispersal rather than vicariance resulting from continental drift.

## 5 CONCLUSION

Here, we inferred the first broadly sampled molecular phylogeny of Dryophthorinae, finding support for some but not all existing taxa. All tribes but Rhynchophorini were found to be monophyletic, but higher support is required to ascertain the paraphyly of Rhynchophorini with more confidence. *Nephius* is closely related to Dryophthorini and Stromboscerini, and there is strong evidence for paraphyly of Sphenophorina. We find a large gap between the divergence of the largely monocot‐specialist Dryophthorinae from their sister group, the fungus associated woody‐dicot feeding Platypodinae at the Jurassic‐Cretaceous boundary and the diversification of extant species in the Cenozoic, highlighting the role of codiversification with the two similarly divergent, main lineages of angiosperms, the monocots and dicots. Future areas of investigation on this group include biogeography of this pantropical group, sequencing more genes to resolve the base the of the tree and revisions of lower‐level taxa suspected to be nonmonophyletic.

## CONFLICT OF INTEREST

The authors declare no conflict of interest.

## AUTHOR CONTRIBUTION

**M. Lourdes Chamorro:** Conceptualization (lead); Data curation (supporting); Formal analysis (equal); Investigation (equal); Methodology (equal); Visualization (equal); Writing‐original draft (lead); Writing‐review & editing (equal). **Bruno de Medeiros:** Conceptualization (supporting); Data curation (lead); Formal analysis (equal); Investigation (equal); Methodology (equal); Visualization (equal); Writing‐original draft (equal); Writing‐review & editing (equal). **Brian D. Farrell:** Validation (equal); Writing‐review & editing (equal).

### OPEN RESEARCH BADGES

This article has earned an Open Data Badge for making publicly available the digitally‐shareable data necessary to reproduce the reported results. The data is available at https://osf.io/537gq/?view_only=6924c0a843af4f8aba167626a7111df7.

## Supporting information

Appendix S1

Appendix S2

Appendix S3

Appendix S4

Appendix S5

Appendix S6

Legends S1

## Data Availability

New sequences are deposited in NCBI Genbank accessions MW306940–MW306958 and MW306920–MW306939. Alignments, code, software outputs and resulting trees in nexus format are available in the github repository https://github.com/brunoasm/dryophthorinae_rRNA with a permanent copy in Figshare (https://doi.org/10.25573/data.12756233).
