# Supplementary figures and images for: First phylogenetic analysis of Dryophthorinae (Coleoptera, Curculionidae) based on structural alignment of ribosomal DNA reveals Cenozoic diversification
Source: Ecol Evol. 2021 Feb 9;11(5):1984–98. doi: 10.1002/ece3.7131 (PMC7920784; doi:10.1002/ece3.7131)

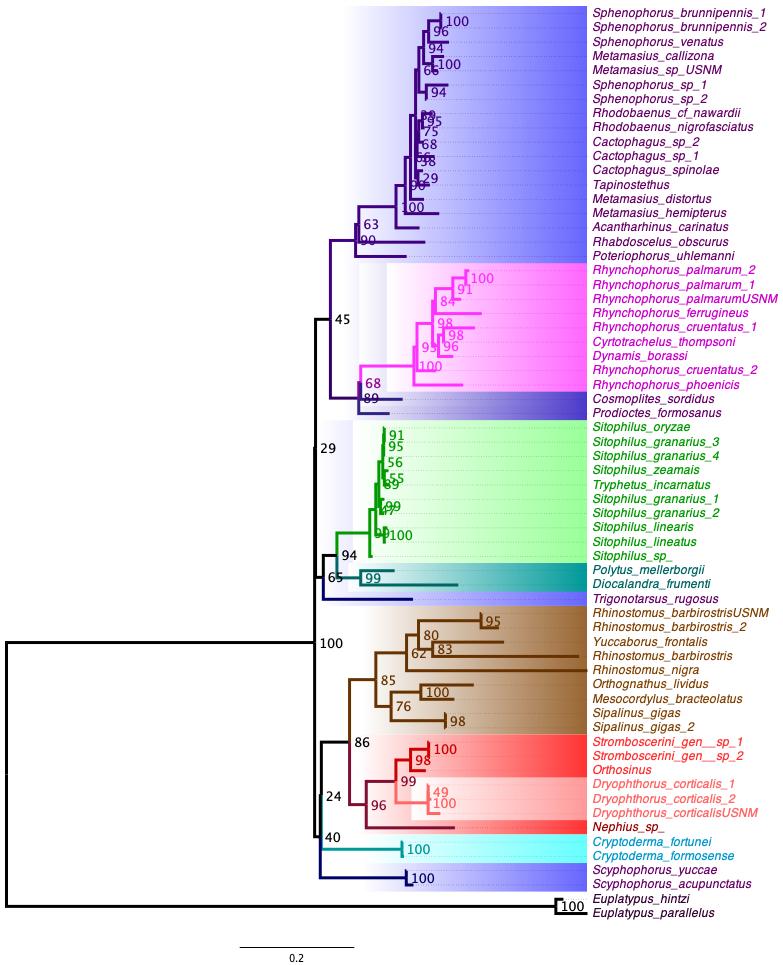

Supplement: Supplementary file 1 — Appendix S1 [file ECE3-11-1984-s001.jpg]

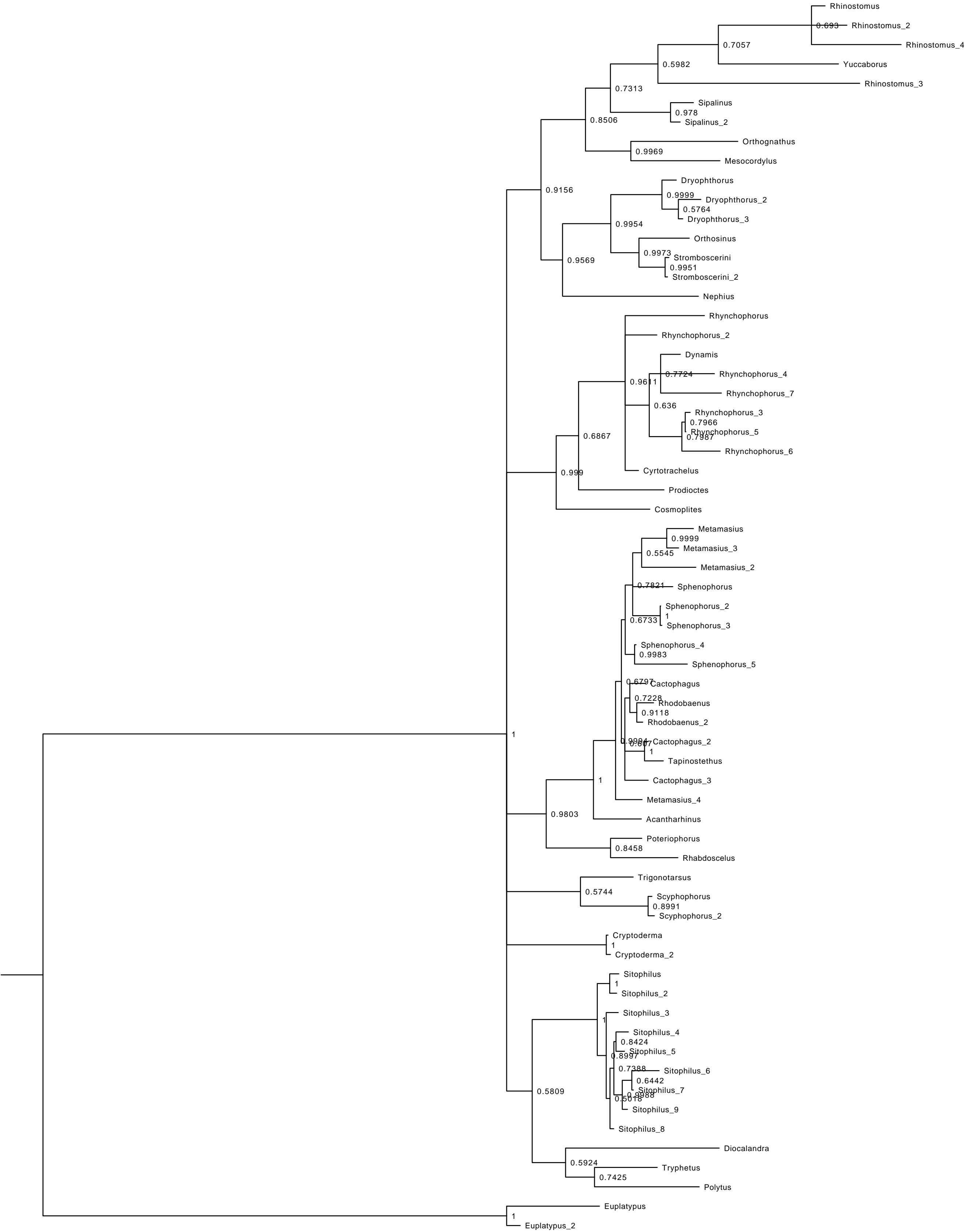

Supplement: Supplementary file 5 — Appendix S5 [file ECE3-11-1984-s005.pdf]

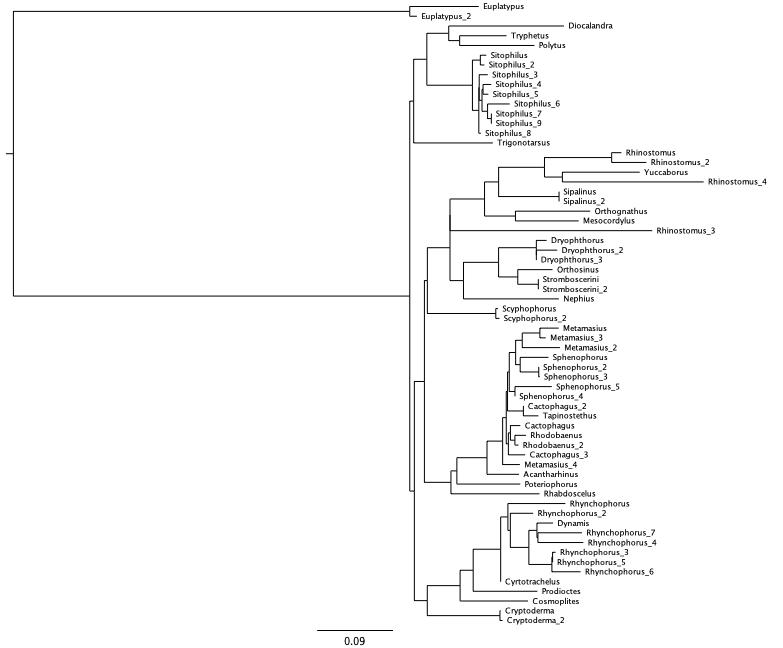

Supplement: Supplementary file 6 — Appendix S6 [file ECE3-11-1984-s006.jpg]
