# Supplementary material for: First phylogenetic analysis of Dryophthorinae (Coleoptera, Curculionidae) based on structural alignment of ribosomal DNA reveals Cenozoic diversification
Source: Ecol Evol. 2021 Feb 9;11(5):1984–98. doi: 10.1002/ece3.7131 (PMC7920784; doi:10.1002/ece3.7131)
Supplement: Supplementary file 4 — Appendix S4 [file ECE3-11-1984-s004.docx]

Appendix S4 – Partitions and substitution models

Substitution models for each partition, with number of sites in partition within parenthesis. See main text for partitioning and model choice methods. Details on the sites covered by each partition can be found in input files for each analysis.

| Alignment | Analysis | partitions |
| --- | --- | --- |
| Structural alignment | PartitionFinder for Mrbayes | SYM+I+G (543)  JC (48)  K80+G (383)  HKY+G (533)  HKY +G (413)  JC (61)  SYM+I+G (1613)  HKY (118)  F81 (52) |
| Structural alignment | IQTREE | TIM3+F+I+G4 (957)  TIM3e+R3 (1661)  K2P+G4 (501)  HKY+F+G4(413)  HKY+F (230) |
| MUSCLE Alignment | PartitionFinder for Mrbayes | SYM+G (1551)  SYM+I+G (2213) |
| MUSCLE Alignment | IQTREE | TIM3e+G4 (1590)  TIM2e+I+G4 (2249) |
