# Supplementary material for: First phylogenetic analysis of Dryophthorinae (Coleoptera, Curculionidae) based on structural alignment of ribosomal DNA reveals Cenozoic diversification
Source: Ecol Evol. 2021 Feb 9;11(5):1984–98. doi: 10.1002/ece3.7131 (PMC7920784; doi:10.1002/ece3.7131)
Supplement: Supplementary file 7 — Legends S1 [file ECE3-11-1984-s007.docx]

Appendix S1 –

Maximum Likelihood topology of Dryophthorinae of the combined 18S and 28S aligned using primary and secondary structure. Numbers indicate support values.

Appendix S2 – Table with Genbank sequences included in this study.

Appendix S3 – Table with USNM voucher numbers and Genbank accessions.

Appendix S4 – Appendix 4 – Partitions and substitution models

Substitution models for each partition, with number of sites in partition within parenthesis. See main text for partitioning and model choice methods. Details on the sites covered by each partition can be found in input files for each analysis.

Appendix S5 ­– Mr. Bayes aligned without using secondary structure.

Appendix S6 ­– IQ Tree aligned without using secondary structure.
